# Genomic Identification of RNA Editing Through Integrating Omics Datasets and the Clinical Relevance in Hepatocellular Carcinoma

**DOI:** 10.3389/fonc.2020.00037

**Published:** 2020-02-14

**Authors:** Juan Chen, Lu Wang, Fangbin Wang, Jian Liu, Zhenyu Bai

**Affiliations:** ^1^School of Food and Biological Engineering, Hefei University of Technology, Hefei, China; ^2^Engineering Research Center of Bio-Process, Ministry of Education, Hefei University of Technology, Hefei, China; ^3^Department of Laboratory, General Hospital of Pingmei Shenma Medical Group, Pingdingshan, China

**Keywords:** RNA editing, hepatocellular carcinoma, post-transcriptional regulation, bioinformatics, prognosis

## Abstract

RNA editing is a widespread post-transcriptional mechanism to introduce single nucleotide changes to RNA in human cancers. Here, we characterized the global RNA editing profiles of 373 hepatocellular carcinoma (HCC) and 50 adjacent normal liver samples from The Cancer Genome Atlas (TCGA) and revealed that most editing events tend to occur in minor percentage of samples with moderate editing degrees (20–30%). Moreover, these RNA editing prefer to be A-to-I RNA editing in protein coding genes, especially in 3′UTR regions. Considering the association between DNA mutation and RNA editing, our analysis found that RNA editing maybe a complementary event for DNA mutation of HCC risk genes in HCC patients. We next identified 454 HCC-related editing sites, and many locate on the same genes with the same editing patterns. The functional consequences of editing revealed 2,086 functional editing sites and demonstrated that most editing in coding regions are non-synonymous variations. Furthermore, our results showed that editing in the 3′UTR regions tend to influence miRNA–target binding, and the editing degree seems to be negatively correlated with gene expression. Finally, we found that 46 HCC-related editing sites with consequence are able to distinguish the prognosis differences of HCC patients, suggesting their clinical relevance. Together, our results highlight RNA editing as a valuable molecular resource for investigating HCC mechanisms and clinical treatments.

## Introduction

Hepatocellular carcinoma (HCC) is a kind of malignant tumor with high mortality. It ranks third among all cancer-related mortality in the world. About 3% of the patients with cirrhosis can result in HCC, which is the most serious complication in chronic liver diseases (1). The high mortality rate of HCC is mainly due to it being asymptomatic in the early stage and the lack of effective treatments for even mid-term patients. This poses a great threat to the patient's life and also brings heavy economic burdens to the society and families. Therefore, it is of great significance to clarify the pathogenesis of HCC as soon as possible, and thus formulate more effective strategies for clinical diagnosis and treatment. Previous studies on the pathogenic mechanisms of cancer suggested that DNA mutations are a driving factor in cancer development; however, many HCC tumor samples were found to be free of carcinogenic DNA-driven mutations (2), which indicate that other driving events are involved in the occurrence and development of HCC.

RNA editing is an important post-transcriptional modification event, which change genetic information at RNA level and generate results similar to DNA mutations, thus increasing the diversity of transcripts and proteomes (3). The most common type of RNA editing in human cells is the transformation of adenine nucleotides (A) into inosine (I), which was mediated by adenosine deaminase family proteins (ADARs) (4). Because inosine (I) is recognized as guanine (G) nucleotide during translation, it is also called A-to-GRNA editing. In addition to changes in A-to-I (G) RNA editing, human cells also have a small number of other RNA editing types (5). Recent bioinformatics analysis found that RNA editing events are extensive across the human transcriptome (6).

RNA editing in normal cells are associated with adaptive evolution and cell development (7). Conversely, dysfunction in RNA editing systems will have a series of effects on subsequent RNA regulation processes. First, RNA editing in the protein coding region can affect amino acid translation, producing proteins with different structures or functions, and then affecting protein expression activity. For example, the A-to-I hyper-editing in RHOQ transcripts will induce the abnormal elevation of RHOQ protein, and thus promote the invasion and metastasis of cancer cells in colorectal cancer (8). The editing of SLC22A3 transcript can induce the down-expression of SLC22A3 protein, which contributes to the early invasion in familial esophageal squamous cell carcinoma (9). Second, about 95% of multi-exon genes produce different transcripts through alternative splicing (10, 11), which is common in human liver cancer (12). RNA editing occurring in splicing sites or splicing regulatory elements possibly affects the variable splicing processes of RNA. For instance, the hyper-editing on one of the potential branching sites of PTPN6 causes the third intron retention, which is associated with the pathogenesis of acute leukemia (13). Third, genome-wide sequencing analysis revealed that most RNA editing sites were located in the non-coding regions of the genome, including the 3′UTR, intron and intergenic regions, which may influence the regulation of non-coding RNAs, especially miRNA regulation on the 3′UTR regions (14, 15). These studies indicate that RNA editing events will affect a series of downstream RNA regulation processes, which are closely related to the disease processes.

Recently, the incidence and progression of HCC were found associated with RNA editing events and may further help us reveal the pathogenic mechanisms underlying HCC. Chen et al. suggested that the hyper-editing event of AZIN1 results in a serine-to-glycine substitution at residue 367 of the AZIN1 protein, which may be a potential driver in the pathogenesis of HCC (16). Chan et al. identified an average of 20,007 A-to-I RNA editing events in transcripts by utilizing RNA-seq of three paired HCC and their adjacent non-tumor samples, then validated the expression level of ADARs that are related to editing degrees of FLNB and COPA in a large cohort with microarray analysis. They also found that the expression level of ADARs which mediate A-to-I RNA editing is related to the risk of HCC recurrence (17). Similarly, research on two pairs of HCC patients revealed that BLCAP transcript is hyper-edited, which will enhance the phosphorylation of AKT, MTOR, and MDM2 and inhibit the function of TP53, thus promote cell proliferation and tumor development (18). Another study identified HCC-related RNA editing sites by genomic and transcriptomic analysis of nine pairs of HCC and normal samples (19). However, the number of HCC patients used in these studies is relatively small, and their statistical efficacy is limited. Furthermore, the main concern is the limited effect of ADAR enzymes on RNA editing, and the limited analysis of downstream regulation systems, which RNA editing may affect.

Recently, The Cancer Genome Atlas (TCGA) project provides a large number of omics data of malignant tumors. Han et al. and Paz-Yaacov et al. identified RNA editing sites in multiple cancer types from TCGA, including HCC (20, 21). However, they focused on pan-cancer analysis and only used a part of the HCC samples. Han et al. focused on existing RNA editing sites annotated in the RADAR database. Moreover, they removed all mutation sites annotated in the COSMIC database and the ones that were not matched to specific tumor samples. Thus, they may miss a number of HCC-related RNA editing events. Paz-Yaacov et al. focused on the clinical influence of Alu-specific RNA editing and just considered 30 pairs of HCC cancer and normal samples. Most importantly, the specific changes in downstream RNA regulation system caused by RNA editing were not thoroughly analyzed, and the genome-wide distribution pattern of RNA editing in HCC is not described in both studies. In addition, as they focused on the common pathogenic mechanism of multiple cancer types, many HCC-related RNA editing sites were not presented. We suggested that it should be clearly described whether the RNA editing was observed in cases where DNA mutations are absent in HCC-relevant driver genes.

In our study, we intend to *de novo* identification of HCC-related RNA editing sites by integrating multiple omics data with bioinformatics methods, including genomic mutation, transcriptomic variation, and reference single nucleotide polymorphism (SNP) information, using 373 HCC tumor samples and 50 adjacent normals from TCGA. Here, the genomic distribution pattern of RNA editing events was described. We also deeply analyzed the influence of HCC-related RNA editing on downstream regulation system, including the effect on protein translation and miRNA regulation. Based on clinical information of HCC patients, our study further identified new biomarkers for clinical prognosis, which will promote the disclosure of molecular mechanisms of HCC from the perspective view of RNA editing.

## Materials and Methods

### Data Retrieval

Pair-end RNA-seq BAM files originating from 373 HCC cancer samples and 50 adjacent normal liver samples were downloaded from the database of Genotypes and Phenotypes (dbGaP) originally from The Cancer Genome Atlas (TCGA, https://portal.gdc.cancer.gov/) research project (22). Validated RNA-seq FASTQ files of HCC cell lines and normal liver samples were downloaded from ArrayExpress (https://www.ebi.ac.uk/arrayexpress/, E-MTAB-4052) (23), including three human normal liver samples and two Huh7 RNA-seq FASTQ datasets. DNA mutation, gene expression, and clinical information datasets were also downloaded from TCGA. Single nucleotide polymorphism (SNP) annotations were downloaded from dbSNP version 137 (24) and the 1000 Genomes Project (25).

Gene annotation of the 3′UTR, 5′UTR, CDS, and intron regions were downloaded from the UCSC table browser (http://genome.ucsc.edu/cgi-bin/hgTables) (26). In addition, functional annotation gene sets were downloaded from the MsigDB database (http://software.broadinstitute.org/gsea/msigdb) (27).

We obtained the list of known tumor suppressive genes (TSGs) and oncogenic genes (OGs) from a previous study (28), which integrates mRNA expression, copy number variations (CNV), and DNA mutation information and generated a continuous ranked list for each gene, ranging from more negative (TSGs) to more positive (OGs) with consistent changes across tumors. Here we used a strict score threshold to get OGs (score ≥7) and TSGs (score ≤−7) for further analysis. The statistical significances for the enrichment of OGs and TSGs were calculated by hypergeometric tests. All of the data resources mentioned above are shown in Supplementary Table S1.

### *De novo* Detection of RNA Editing Sites

First, the downloaded BAM files of the TCGA samples were converted to FASTQ using BEDtools (29), the FASTQ files were aligned to the human reference genome (GRCh38) by STAR with default parameters (--outFilterMultimapScoreRange 1 –sjdbScore 2 –outFilterScoreMinOverLread 0.33 --outFilterMultimapNmax 20 --sjdbOverhang 100) (30). Second, putative RNA editing sites were identified by Genome Analysis Toolkit (GATK4) with default parameters (HaplotypeCaller --gcs-max-retries 20 --heterozygosity 0.001 --max-reads-per-alignment-start 50 --min-base-quality-score 10), using uniquely mapped reads after PCR duplicates were removed (31). Third, computational filters for *vcf* files were applied through five steps: (i) removing DNA mutation sites for each HCC sample; (ii) taking out all known SNPs in dbSNP version 137 or the 1000 Genome Project, and also insertion or deletion sites; (iii) further filtering sites to obtain editing sites with high confidence: if Fisher Strand (FS) >20, or Quality by Depth (QD) <2, or editing was supported <2 reads, or total coverage reads <10, these sites were removed, and we required at least 10% difference between the editing degrees of 90% quantile and 10% quantile across all samples; (iv) sites with 100% editing degree were also filtered, as 100% editing efficiency is thought to be unrealistic (6); (v) keeping variants detected in at least 1% of the samples because they are unlikely to be rare variants. Editing degree was defined as the percentage of edited reads among the total mapped reads at a given site (20).

Finally, we restricted editing sites to 46 human chromosomes. To get the exact RNA, which was edited, we used BEDtools to map the editing sites with gene annotation *gtf* files. If the RNA editing sites were simultaneously mapped to two strands, these sites were further removed. At last, we get 19,431 RNA editing sites for further analysis.

The validated RNA-seq FASTQ datasets were aligned to the human reference genome (GRCh38) by *bowtie*, processed by GATK4 with default parameters, with no matched DNA mutation datasets. Other pipelines were similar to the aforementioned method.

### Identification of HCC-Related RNA Editing Sites

HCC-related RNA editing sites include HCC gain, HCC loss, and significant dysregulated editing (dys-edit) sites. HCC gain or HCC loss editing sites were defined by Fisher's exact test (Benjamini–Hochberg correction, adjust *p* < 0.05), with HCC gain editing sites restricted to be no more than 5% editing sites in normal samples and HCC loss editing sites restricted to be no more than 5% editing sites in cancer samples. Dys-edit sites were just focused on the editing degree of 50 HCC cancer samples and mapped 50 normal samples, which were determined by two steps: (i) using the paired Student's *t*-test (Benjamini–Hochberg correction, adjust *p* < 0.2 and *p* < 0.01); (ii) restricting editing sites to those having more than 0.25 editing degree change in at least two pairs of normal and cancer samples. The HCC dys-edit sites identified in the above two steps were changed in the same direction (simultaneously hyper-edited or hypo-edited in two steps). Finally, we identified 454 HCC-related RNA editing sites.

### Functional Enrichment Analysis for HCC-Related RNA Editing Sites

First, HCC-related RNA editing sites were mapped to gene name by BEDtools. Then, we performed functional enrichment analysis by hypergeometric test (Benjamini–Hochberg correction, adjust *p* < 0.05). We focused on chemical and genetic perturbations (CGP), reactome pathways and biological processes (BP) of gene ontology (GO) originating from MsigDB.

### The Functional Consequence Analysis for RNA Editing Sites

#### Protein Coding and Alternative Splicing Change

To define whether an editing site can change protein translation or alternative splicing, we re-annotated them by ANNOVAR (32).

#### miRNA–Target Binding Prediction

RNA editing in the 3′UTR regions may influence the binding and regulation of miRNAs, including loss of existing miRNA–target regulation and gain of new regulation, or change the binding strength of existing miRNA–target regulation. Therefore, for each RNA editing site in the 3′UTR regions, we simultaneously calculated and compared the miRNA binding in both edited and reference 3′UTR regions. We computationally predicted whether an miRNA binds to the mRNA regions around editing sites using miRanda (33) as described in Hwang et al. (5), which calculates the binding energy to estimate the thermodynamic properties of a predicted duplex and a complementarity score to estimate the mismatch. Briefly, for mRNA as a target sequence, two types of sequences were prepared with flanking regions of editing sites (50 bp upstream and downstream) in all mRNA transcripts in UCSC: the reference sequence and the editing sequence. The mature miRNA sequences were also prepared, which were obtained from miRBase (34). The binding energies between miRNA sequence with both a reference and edited mRNA sequences were calculated by miRanda (v3.3a) with default parameters (score > 140, gap-open penalty set to −4 and gap-extend penalty set to −9). We next used delta G < -14 kcal/mol as a threshold for free energy of duplex formation to obtain more confident miRNA–target regulations, which were maintained for the following analysis. The comparison was performed for these two types of mRNA sequence, with all the predicted binding pairs of miRNAs and mRNA targets. If miRNA–target relationships just appeared in reference but not in the edited sequence, these were defined as “edited loss” conversely, defined as “edited gain.” As for the relationships that both appeared in the reference and edited sequences, if the binding energies changed more than 14 kcal/mol between the reference and edited sequences, they were defined as “edited change.” The “edited gain,” “edited loss,” and “edited change” constituted the candidate pool of RNA editing sites that may induce miRNA–target regulations to change. Finally, if the editing of an mRNA target induces more than 10 miRNA–target regulation change, this editing site was defined as having an miRNA–target regulation consequence.

#### Expression-Related RNA Editing Sites

To define whether RNA editing can influence RNA expression, we calculated the Pearson correlation between editing degree and expression level among samples where both expression and editing degree were measured (editing degree is not 0; Benjamini–Hochberg correction; adjust *p* < 0.05). The normalized gene expression levels of raw read counts were calculated using DESeq2 (35).

#### Correlation Between Editing Risk Score and Survival

First, we defined the editing risk score for each HCC clinical sample. Univariate Cox regression analysis was performed to evaluate the association between survival duration and the editing degree of each editing site. A regression coefficient with a plus sign indicated that increased editing degree is associated with an increased risk of survival (risky editing); conversely, a minus sign indicated that an increased editing degree is associated with a decreased risk of survival (protective editing). More specifically, for editing sites, which are located in a gene region, we assigned a risk score to each HCC patient according to a combination of the product of the editing degree and gene expression, weighted by 1 or −1 according to the regression coefficients from the univariate Cox regression analysis mentioned above. The risk score for each patient was calculated as follows:

$$\begin{array}{l} Risk\text{\_}Score=\overset{n}{\underset{i\;=\;1}{\sum }}\beta_{i}\;*\;Exp_{gene\left(i\right)}\;*\;Edit_{i} \end{array}$$

where, β_*i*_ is 1 or −1 when the Cox regression coefficient of the editing site *i* is a positive or negative value, respectively. The *n* is the number of HCC-related RNA editing sites with consequence that are located in gene regions with an editing degree more than 0 in each sample. *Exp*_*gene*(*i*)_ is the expression level of gene in which the editing site *i* occurs. *Edit*_*i*_ represents the editing degree of the editing site *i*.

To investigate whether risk scores are associated with tumor grades and tumor stages, we use the Wilcoxon rank-sum test. As the sample number with a tumor grade 4 or tumor stage 4 is too small, only 12 and 13, respectively, we only considered grade 1, grade 2, and grade 3 plus (including grade 3 and grade 4) for tumor grades and stage 1, stage 2, and stage 3 plus (including stage 3 and stage 4) for tumor stages. All patients were then classified into high-risk and low-risk groups using the median risk score as the cutoff point. The Kaplan–Meier method was further used to estimate the differences in overall survival time for these two patient groups (log-rank test). Patients having higher risk scores were expected to have poor survival outcomes.

We further focused on three prognostic-related editing sites whose editing degrees are significantly correlated with survival (*p* < 0.05). The risk scores were calculated according to the mathematical formula above. For HBV/HCV infected patients or non-alcoholic fatty liver patients, we did a similar analysis mentioned above to obtain a risk score for each sample using three prognostic-related editing sites.

To determine whether editing risk scores can provide additional predictive power, we performed a multivariate survival analysis using prognostic factors, including gender, age, body mass index (BMI), grade, and stage of HCC patients, along with editing risk score.

## Results

### Global Properties of the Inferred RNA Editing Sites in HCC and Normal Samples

**Figure 1:**
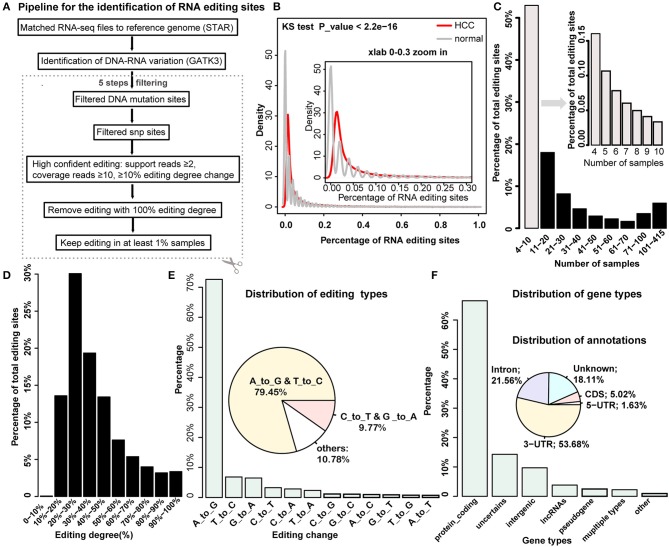
Systemic identification and global properties of RNA editing sites in hepatocellular carcinoma (HCC) and normal samples. **(A)** The pipeline for the identification of RNA editing site in HCC and normal samples. **(B)** The distribution of the percentage of RNA editing sites in HCC cancer and normal sample. **(C)** The distribution of editing percentages across different intervals of samples. **(D)** The percentage of editing sites across different intervals of editing degrees. **(E)** The distribution of editing variation types. **(F)** The distribution of gene types and genomic annotations for identified RNA editing sites.

Several *de novo* methods for detecting RNA editing were developed recently (5, 6, 20). Here we combined these methods and proposed a multi-stage method to gradually identify RNA editing sites by integrating DNA mutation and SNP datasets (Figure 1A) (see details in the Methods section). Totally, we obtained 19,431 RNA editing sites for further analysis. In general, HCC tumor samples have a higher percentage of RNA editing sites compared to the normal samples (Figure 1B). More than half of the editing sites occurred in no more than 10 samples (Figure 1C). Most editing sites presented moderate editing degree, where 20–30% editing degree accounted for the largest proportion (Figure 1D). We also found that A-to-IRNA editing accounts for most of the RNA variants in the list, and the following enriched variant types were T-to-C, G-to-A, and C-to-T RNA editing, which was consistent with previous research [Figure 1E; (5, 6)]. We next annotated these editing sites with gene types and revealed that most of the editing sites are located on protein-coding genes. In addition, a moderate number of RNA editing sites are located in intergenic and lncRNA regions (Figure 1F). Consistent with a previous study, more than half of the editing sites are in the 3′UTR regions, accounting for 53.68%, and the following are in the intron regions (Figure 1F). Notably, Han et al. reported just a few A-to-I RNA editing in the CDS regions; here we identified about 5% editing events in the CDS regions, which include many other types of RNA editing. After we restricted to A-to-I RNA editing, the percent decreased to be 1.66% in the CDS regions (Supplementary Figure S1).

### RNA Editing May Be a Complementary Event for DNA Mutation of HCC Risk Genes in HCC Patients

**Figure 2:**
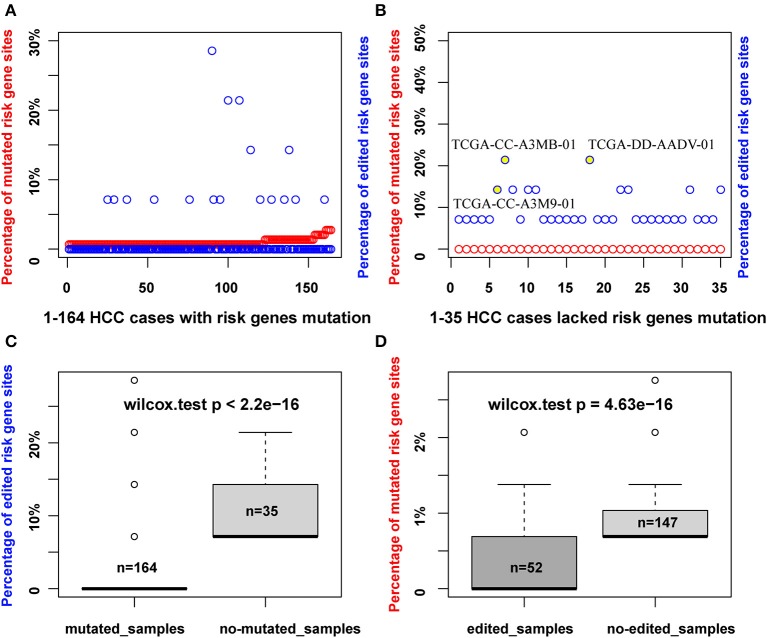
RNA editing maybe a complementary event for DNA mutation of HCC risk genes in HCC patients. **(A)** The percentage of mutated sites or edited sites in 164 HCC samples with DNA mutation of HCC risk genes (red points, number of mutated sites/total mutated HCC risk sites identified; blue points, number of edited sites/total edited HCC risk sites identified). **(B)** The percentage of mutated sites or edited sites in 35 HCC samples without DNA mutation of HCC risk genes. **(C)** The percentage of edited sites account for total edited HCC risk sites identified in mutated and non-mutated HCC samples. **(D)** The percentage of mutated sites account for total mutated HCC risk sites identified in edited and non-edited samples.

As RNA editing and DNA mutation have a similar effect to increase the diversity of transcripts in cells, thus we wonder whether there is a connection between these two events. For a given gene region, if it is identified to be mutated, it would not be identified as editing at the same time in our recognition method. However, we can dissect whether there is a correlation between RNA editing and DNA mutation in HCC risk gene sets. We obtained 33 HCC relevant cancer genes from a previous report (36), which we defined as HCC risk genes, such as TP53, PTEN, and TERT, of which we observed 24 risk genes with DNA mutation (145 mutation sites involved), and six risk genes with RNA editing (14 editing sites involved). HCC patients with DNA mutation (164) and 35 cases lacking DNA mutations in HCC risk genes were observed (Figures 2A,B). It is found that all of these 35 patients that lacked DNA mutation had RNA editing in the HCC risk genes. For example, patient TCGA-CC-A3M9-01 had no mutated risk genes, but had RNA editing sites in PTEN (chr10:87967417) and UBE2H (chr7:129830735). In patient TCGA-CC-A3MB-01, three sites of RNA editing occurred in the risk gene, TERT (chr5:1262789, chr5:1262852, chr5:1263350), and the same with patient TCGA-DD-AADV-01 (Figure 2B). In addition, we found that the percentage of the edited sites in the HCC risk genes is much higher in patients who lacked DNA mutation compared to patients with DNA mutation in the HCC risk gene regions (*p* < 2.2e-16, Wilcoxon test) (Figure 2C). Meanwhile, the mutation percentage is much lower in edited patients compared with non-edited patients in HCC risk gene regions (*p* = 4.63e-16, Wilcoxon test) (Figure 2D). The observations above implied that RNA editing was a risk factor to HCC as a complementary event for DNA mutation in HCC risk genes.

### HCC-Related RNA Editing Prefer to Locate on Liver-Specific Genes

**Figure 3:**
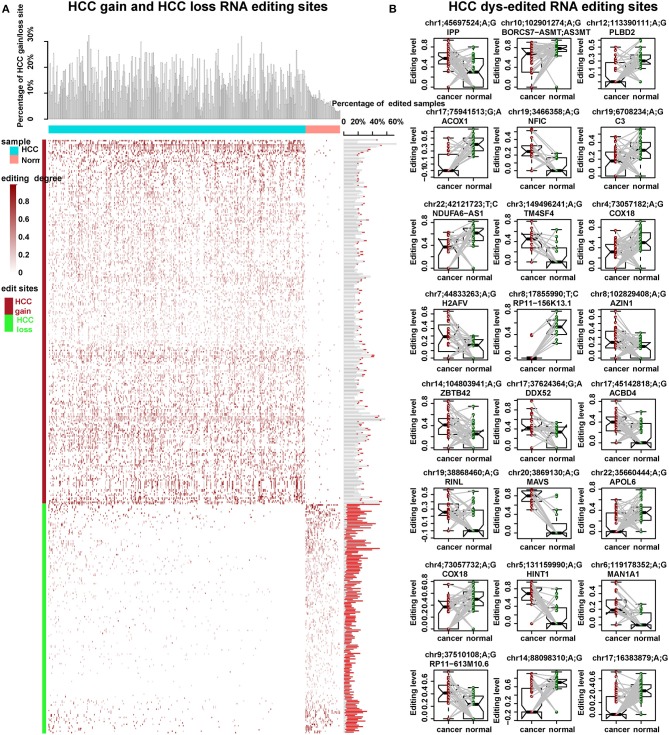
HCC-related RNA editing sites (454) were identified. **(A)** HCC gain and HCC loss RNA editing sites across 373 HCC cancer samples and 50 normal samples. Upper: the percentage of HCC gain and loss site in each HCC or normal samples. Middle: the editing degree in each HCC or normal cases. Right: the percentage of edited samples for each HCC gain or loss editing site. The box filled in red showed the percentage of edited samples in normal samples. Meanwhile, the one filled in white showed the percentage of edited samples in HCC cancer samples. **(B)** The editing degrees of 24 HCC dys-edited RNA editing sites in 50 paired HCC cancer and normal samples. The last two are in the gene interstitial regions.

Chen et al. proved that RNA editing of AZIN1 promotes progression of HCC (16). In our study, by genome-wide identification of RNA editing sites across large RNA-seq samples, we aimed to reveal more HCC-related RNA editing sites. Generally, we identified 454 HCC-related RNA editing sites, including 264 HCC gain, 166 HCC loss, and 24 HCC dys-edited sites (Figures 3A,B, Table 1). Notably, a serine-to-glycine substitution at residue 367 of AZIN1 was also identified by our method, which is reported to be hyper-edited in HCC patients compared to normal samples in previous studies (16). Two editing sites of COX18 were hypo-edited in HCC compared with paired normal samples. The most significantly dys-edited RNA editing sites were editing on the ACOX1 (chr17: 75941513, *p* = 1.15e-05). Notably, ACOX1 was reported to play important roles in cancer development of HCC by stimulating hepatic fatty acid oxidation and H_2_O_2_ accumulation (37).

**Table 1:** HCC-related RNA editing sites.

	**Number of editing sites in protein-coding genes**	**Number of involved protein-coding genes**	**Total number of RNA editing sites**
HCC gain	208	134	264
HCC loss	93	67	166
Dys-edited	19	18	24
Total	320	213	454

To further investigate the biological function of these HCC-related RNA editing sites, we performed functional enrichment analysis for these genes with the HCC-related RNA editing sites (hypergeometric test, adjust *p* < 0.05; Supplementary Figure S2, Supplementary Table S2). Our results indicated that HCC-related editing genes prefer to be liver-specific genes, which were involved in nuclear transport, catabolic and cell cycle processes. The enrichment of liver-specific genes and liver cancer-associated genes suggested that RNA editing can be a potential research area to analyze the mechanism, clinical prevention, and treatment of HCC patients.

### HCC-Related Editing Sites Prefer to Locate on the Same Genes With the Same Editing Patterns

**Figure 4:**
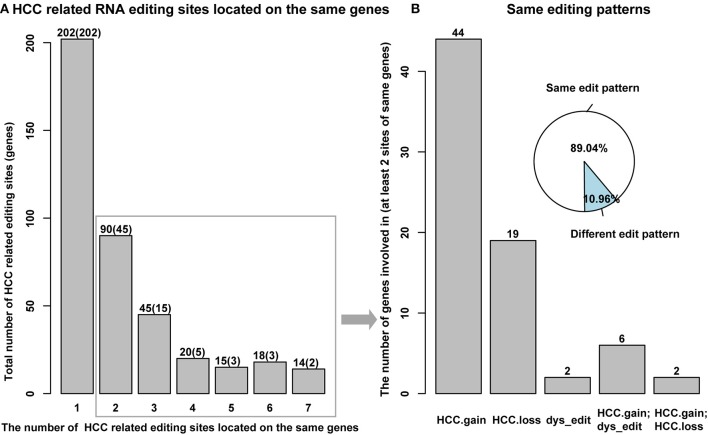
HCC-related RNA editing sites prefer to locate on the same genes with same editing patterns. **(A)** Several HCC-related RNA editing sites located on the same genes. Y axis means the total number of editing sites, and the number of genes involved in were shown in parenthesis. X axis means the number of different editing sites located on the same genes. **(B)** Different HCC-related RNA editing sites, which located on the same genes prefer to have the same editing patterns.

A large percent of HCC-related RNA editing sites were located on gene regions (88.99%, 404/454) especially for HCC gain and dys-edited sites. Interestingly, 73 genes were shared by at least two HCC-related RNA editing sites and accounted for 50% (202 editing sites of 404) (Figure 4A, Supplementary Table S3). Notably, we found that different editing sites located on the same genes tend to be with the same HCC editing type, with few exceptions (Figure 4B, Table 2). For example, there are seven HCC-related RNA editing sites located on MDM4; meanwhile, these seven sites are all HCC gain editing patterns. About 89.04% genes with different editing sites have the same editing patterns, which indicated that these editing sites may have important roles in HCC initiation and development.

**Table 2:** Several HCC-related RNA editing sites located on the same genes with the same editing patterns.

**Gene**	**Frequency**	**HCC-related sites**
MDM4	7	7 HCC gain
DHODH	7	7 HCC loss
JRK	6	6 HCC gain
GINS1	6	6 HCC gain
ALDH2	6	6 HCC loss
SPC24	5	5 HCC gain
SERPINF2	5	5 HCC gain
APC2	5	5 HCC gain
ZNF517	4	4 HCC gain
MOGAT2	4	4 HCC loss
MAVS	4	3 HCC gain; 1 HCC dys-edited
HINT1	4	3 HCC gain; 1 HCC dys-edited
ZNF814	3	3 HCC gain
TTC9C	3	2 HCC gain; 1 HCC dys-edited
TRIM56	3	2 HCC gain; 1 HCC dys-edited
RP5-1061H20.4	3	3 HCC gain
POLR1A	3	3 HCC gain
NPLOC4	3	3 HCC gain
MOGAT3	3	3 HCC gain
METTL7A	3	3 HCC gain
IPP	3	2 HCC gain; 1 HCC dys-edited
HAVCR2	3	3 HCC gain
HAMP	3	3 HCC loss
FADS2	3	3 HCC gain
DCAF16	3	3 HCC gain
ADAMTS13	3	3 HCC loss

*This table showed genes related to at least three editing sites*.

### The Functional Consequence of RNA Editing Sites

**Figure 5:**
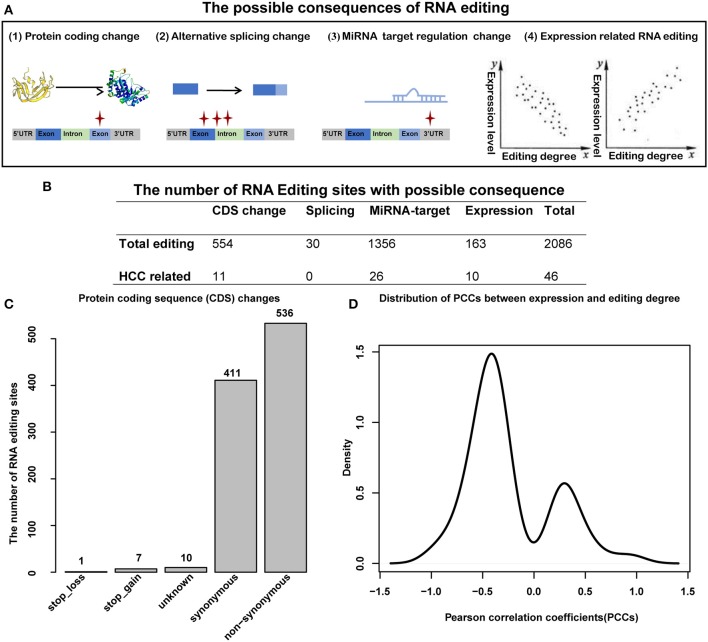
Functional consequence analysis of RNA editing sites. **(A)** The possible functional consequence of RNA editing, including protein-coding sequence change, alternative splicing change, miRNA–target regulation change, and expression level change. **(B)** The number of RNA editing sites with possible consequence. **(C)** The number of RNA editing sites for protein-coding sequence changes. **(D)** The distribution of Pearson correlation coefficients (PCCs) between editing degrees and the expression of corresponding genes.

Previous studies have suggested that RNA editing can be functional and involved in carcinoma initiation and progression by influencing amino acid encoding, alternative splicing, miRNA–target regulation, or expression changing of the corresponding gene [(38, 39); Figure 5A]. Totally, we identified 2,064 editing sites with functional consequence, of which 46 were HCC-related (Figure 5B, Supplementary Table S4). For editing in coding sequence (CDS) regions, we identified 554 editing sites with functional change, including one stop loss, seven stop gain, 10 unknown, and 536 non-synonymous (Figures 5B,C), of which 11 editing sites were HCC related. Specifically, the serine-to-glycine substitution at residue 367 of AZIN1 was reported in a previous study; the editing may induce a conformational change and a cytoplasmic-to-nuclear translocation, which will result in tumor initiation and aggressive tumor progression (16). Another example is serine-to-threonine substitution at residue 1,768 of MUC6, which located in a proximate repeat region annotated in the Uniprot database (40). Importantly, abnormal expression of MUC6 was reported to be associated with many gastrointestinal cancers, such as HCC and cholangiocarcinoma (41). Hence, we supposed that several RNA editing located in important protein domains, which change the properties of the protein, might play roles in the progression of HCC. In terms of alternative splicing, 30 editing sites identified by ANNOVA can induce splicing change, of which no one was HCC related. As more than half of the RNA editing sites located on the 3′UTR regions, next, we considered miRNA–target regulation change caused by RNA editing in the 3′UTR regions. We use miRanda to calculate the binding energies between miRNA sequence and editing sequence (or reference sequence). We identified 1,356 editing sites that affect miRNA–target regulation, of which 26 were HCC related. Among these 26 HCC-related RNA editing sites, the top three affected miRNAs were miR-17-3p, miR-20b-3p, and miR-593-3p (with 11, 7, and 7 target regulation changes induced by HCC-related editing sites, respectively (Supplementary Table S5). Notably, miR-17-3p, miR-20b-3p, and miR-593-3p are widely involved in caner initiation and progression, including HCC (42–46). We observed that the expression levels of 163 RNA editing sites were correlated with the editing degree of corresponding genes, of which 10 were HCC-related editing sites. Moreover, Pearson correlation coefficients (PCCs) tend to be negative values (Figure 5D), which indicated that most of the RNA editing sites might cause downexpression of the corresponding genes.

### HCC-Related RNA Editing Sites With Functional Consequence Can Predict Clinical Prognosis

**Figure 6:**
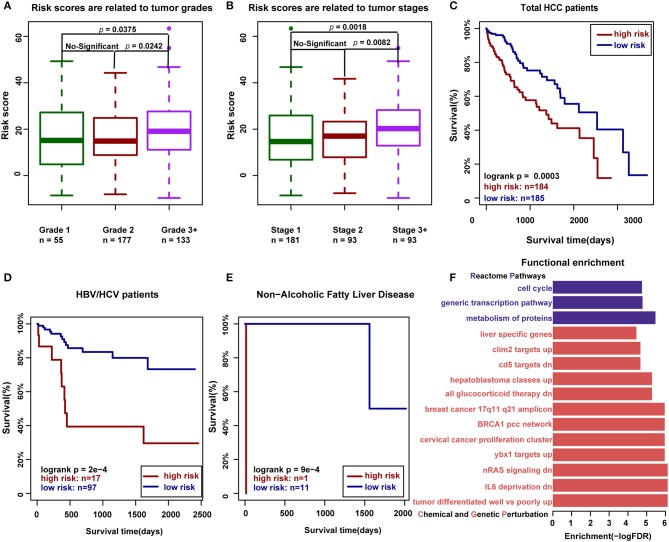
The HCC-related RNA editing sites with functional consequence can predict clinical outcomes of HCC patients. **(A)** Risk scores measured by 46 HCC-related RNA editing sites with functional consequence are related to tumor grades in HCC patients (Wilcoxon rank-sum test). **(B)** Risk scores measured by 46 HCC-related RNA editing sites with functional consequence are related to tumor stages in HCC patients (Wilcoxon rank-sum test). **(C)** Risk scores measured by 46 HCC-related RNA editing sites with functional consequence can predict the prognosis in HCC patients. **(D)** Risk scores measured by three HCC-related prognostic editing sites with functional consequence can predict the prognosis in HBV-/HCV-infected HCC patients. **(E)** Risk scores measured by three HCC-related prognostic editing sites with functional consequence can predict the prognosis in HCC patients with non-alcoholic fatty liver disease. **(F)** Functional enrichment of 46 HCC-related RNA editing sites with consequence. The functional gene sets were downloaded from MsigDB, including Chemical and Genetic Perturbation in red and Reactome pathway in purple.

To further investigate the relationship between RNA editing and HCC clinical characteristics, we first defined the risk score for each clinical sample by integrating the expression levels and editing degrees of HCC-related RNA editing sites with functional consequence, weighted by 1 or −1 according to the regression coefficients from univariate Cox regression analysis (detailed in the Materials and Methods section). As expected, we found that the risk scores measured by 46 HCC-related RNA editing sites with functional consequence correlated with tumor grades and stages in HCC patients, where higher risk scores in patients implicated higher tumor malignancy (higher tumor grades or tumor stages, Wilcoxon rank-sum test) (Figures 6A,B). We further classified all HCC patients into high-risk and low-risk groups using the median risk score as the cutoff point. Our analysis suggested that patients having higher risk scores were expected to have poor overall survival times (log-rank test, *p* = 0.0003; Figure 6C). The risk score was still significantly associated with patient overall survival (hazard ratio = 1.03, *p* = 0.01) in the Cox multivariate analysis, after adjusting for patients' gender, age, BMI, tumor grades, and stages (Table 3), which indicates that these HCC risk RNA editing sites could be potential biomarkers to predict clinical outcomes of HCC patients.

**Table 3:** The results of multivariate survival analysis of 46 HCC-related RNA editing sites with consequence.

	**HR (95% CI)**	***p*-Value**
**Risk score**	1.03 (1.01, 1.04)	0.010^**^
**Gender**		
Female	1 (reference)	
Male	0.90(0.55, 1.45)	0.652
**Ages**	1.00(1.00, 1.00)	0.025^*^
**BMI**	1.03(1.00, 1.06)	0.097
**Grades**		
Grade G1	1 (reference)	
Grade G2	1.46 (0.65, 3.22)	0.369
Grade G3	1.74(0.75, 4.06)	0.196
Grade G4	4.91(1.47, 16.39)	0.010^*^
**Stages**		
Stage T1	1 (reference)	
Stage T2	1.31 (0.71, 2.43)	0.385
Stage T3	1.70 (0.92, 3.13)	0.089
Stage T4	3.83 (1.45, 10.07)	0.007^**^

Values of p < 0.05 were bolded, of which

* p < 0.05 and

** *p < 0.01*.

We next identified three prognostic-related editing sites whose editing degrees are significantly correlated with survival time using univariate Cox regression analysis (*p* < 0.05; Table 4). The median risk score using these three prognostic editing sites and matched genes can also significantly classify patients into separate groups with different clinical outcomes (log-rank test, *p* = 0.03, data not shown). After adjusting for patients' gender, age, BMI, tumor grades, and stages, the risk score still has predictive power (hazard ratio = 1.19, *p* = 0.001; Table 5). As HBV/HCV infection, alcoholic consumption, and non-alcoholic fatty liver diseases are the three main risk factors to HCC patients, we next examined the predictive power of these three editing sites with these risk factors. We found that these three prognostic-related editing sites can be used to predict the clinical outcomes for HBV-/HCV-infected and non-alcoholic fatty liver disease patients (log-rank *p*-values were 2e-4 and 9e-4, respectively) (Figures 6D,E), but not alcoholic consumption patients (data not shown).

**Table 4:** Three prognostic HCC-related RNA editing sites with functional consequence.

**RNA editing**	**Gene types**	**Gene name**	**Editing type**	**Function**
chr12; 111811793;A;G	Protein coding	ALDH2	HCC loss	miRNA–target
chr9;41929326; C;T	Protein coding	CNTNAP3B	HCC gain	CDS change
chr9;65675990; T;G	Protein coding	CBWD5	HCC loss	CDS change

**Table 5:** The results of multivariate survival analysis of three prognostic HCC-related RNA editing sites with consequence.

	**HR (95% CI)**	***p*-Value**
**Risk score**	1.19 (1.07, 1.32)	0.001^**^
**Gender**		
Female	1 (reference)	
Male	0.84 (0.51, 1.38)	0.495
**Ages**	1.00 (1.00, 1.00)	0.036^*^
**BMI**	1.04 (1.01, 1.07)	0.009^*^
**Grade**		
Grade G1	1 (reference)	
Grade G2	1.42 (0.64, 3.15)	0.390
Grade G3	1.88(0.82, 4.28)	0.135
Grade G4	6.64 (2.03, 21.76)	0.002^**^
**Stage**		
Stage T1	1 (reference)	
Stage T2	1.12 (0.60, 2.09)	0.719
Stage T3	1.59 (0.87, 2.91)	0.132
Stage T4	3.24 (1.20, 8.69)	0.020^*^

Values of p < 0.05 were bolded, of which

* p < 0.05 and

** *p < 0.01*.

Furthermore, we dissected the function of these 46 HCC-related RNA editing sites with functional consequence. It is shown that they were widely involved in three reactome pathways: “metabolism of proteins,” “generic transcription pathway,” and “cell cycle” (Figure 6F, Supplementary Table S6). The functional enrichment analysis for chemical and genetic perturbation datasets indicated that they were enriched in tumor differentiation, nRAS signaling pathway, and liver cancer-associated genes.

## Discussion

HCC is a complex disease with poor prognosis and affected by multiple genetic alterations. DNA mutations are the most widely investigated driver events in all cancer types, including HCC. However, the mutation events are not presented in all HCC patients. Furthermore, we found that 98.51% mutation sites occurred in only a single HCC patient. Similar to the consequence of DNA mutation, RNA editing can also produce nucleotide variations at the RNA level. Importantly, the RNA editing seems to be a more sophisticated regulation, as the editing degree can be 0–100%, while DNA mutation should be mutation or not (0 or 1). Here we systematically identified RNA editing sites by integrating DNA mutation and SNP datasets, and found that HCC samples have a significantly high percentage of RNA editing sites compared with normal samples. We also observed RNA editing in 35 cases that lacked DNA mutations in HCC risk genes, and more edited risk genes occurred in mutated patients compared with patients who do not have any DNA mutation in the HCC risk gene. Thus, we proposed that RNA editing may be a risk factor to HCC, as a complementary event for DNA mutation in HCC risk genes.

To further characterize the inferred RNA editing sites in our study, we compared the overlap of A-to-IRNA editing sites in gene regions identified by our study and the editing sites collected in the database of RADAR (Supplementary Figure S3). We found that most A-to-IRNA editing sites were in the RADAR database and accounts for 87.11% (9,565/10,981). Furthermore, we re-identified RNA editing sites in three human normal liver tissues and two HCC cell line samples, and found that 38.11% HCC-related RNA editing sites were edited in at least one sample (173/454), and 39.13% HCC-related editing sites with functional consequence were identified (18/46). These results indicated that RNA editing sites identified by our research were authentic, which can be used for further analysis. Next, HCC-related RNA editing sites were identified by comparing editing in HCC cancer samples and normal samples. Functional annotation showed that HCC-related editing sites prefer to be liver-specific genes, which suggested the important roles of RNA editing in the development of HCC.

Previous studies focused on the effect of ADAR enzymes on RNA editing. Here we addressed another important question on whether these editing sites have an effect on the downstream regulation system. By considering the influence of RNA editing on coding sequence change, alternative splicing, miRNA–target regulation, and expression change, we identified 2,064 editing sites with functional consequence, which accounts for 10.62% of the total RNA editing sites (2,064/19,431). This percentage indicates that RNA editing events may play other important roles, such as the alteration of RNA-binding abilities, which were induced by RNA structure change, which was, in turn, induced by editing. Notably, we found a number of editing sites with miRNA–target regulation changes. More were identified when we set lower thresholds (Supplementary Table S7). If we defined at least one miRNA relationship change by RNA editing, 90.81% editing sites in the 3′UTR regions can induce miRNA–target regulation change (7,651/8,425). Therefore, we should pay more attention to the effect of miRNA–target regulation change induced by RNA editing. Importantly, in regard to the tumor suppressive genes (TSGs) and the oncogenic genes (OGs) reported (28), we demonstrated that these functional HCC-related editing genes were significantly enriched in OGs, but not TSGs (data not shown), and it remains true when we set lower thresholds to identify editing sites with miRNA–target regulation changes (Supplementary Table S7).

Importantly, we found 46 HCC-related RNA editing sites with functional consequence that can be used to predict the clinical outcome in HCC patients. In addition, they have independent predictive power after considering the gender, age, BMI, tumor grades, and stages. We identified three clinical prognostic-related editing sites, which can also provide predictive values in HBV-/HCV-infected patients and non-alcoholic fatty liver disease patients, including editing sites in ALDH2 (chr12:111811793, A-to-I RNA editing), CNTNAP3B (chr9:41929326, C-to-T RNA editing), and CBWD5 (chr9:65675990, T-to-G RNA editing), of which ALDH2 is an HCC-related editing gene with high frequency (six HCC loss editing sites locates on the ALDH2 gene region). Interestingly, multiple studies revealed that ALDH2 is highly correlated with the pathogenic mechanism, risk, and survival of liver cancer patients, including HCC (47–50). Functional annotation suggested that these HCC-related editing sites with functional consequence are widely involved in liver cancer-associated genes, especially tumor suppressive genes, and cancer-associated pathways. Therefore, we assume that some RNA editing events may be “driver events” that promote cancer initiation and progression, as well as play a critical role in clinical survival in cancer patients.

## Data Availability Statement

The datasets analyzed in this study can be found in the database of Genotypes and Phenotypes (dbGaP) originated from The Cancer Genome Atlas (TCGA, https://portal.gdc.cancer.gov/) and ArrayExpress (https://www.ebi.ac.uk/arrayexpress/, E-MTAB-4052).

## Author Contributions

ZB and JL conceived the study. ZB and JC processed the sequencing data. LW and FW analyzed the data. JC wrote the manuscript.

### Conflict of Interest

The authors declare that the research was conducted in the absence of any commercial or financial relationships that could be construed as a potential conflict of interest.
