# Supplementary material for: Genomic Identification of RNA Editing Through Integrating Omics Datasets and the Clinical Relevance in Hepatocellular Carcinoma
Source: Front Oncol. 2020 Feb 14;10:37. doi: 10.3389/fonc.2020.00037 (PMC7033493; doi:10.3389/fonc.2020.00037)
Supplement: Supplementary file 1 [file Data_Sheet_1.zip › Supplementary_files/Supplementary_Material.pdf]

Supplementary Material

Supplementary Figures

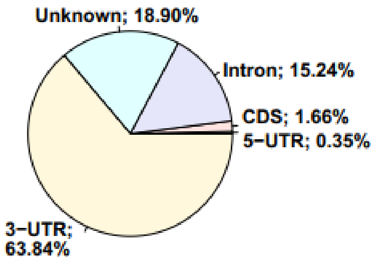

Supplementary Figure S1. The distribution of gene types and genomic annotations for identified A-to-I RNA editing sites.

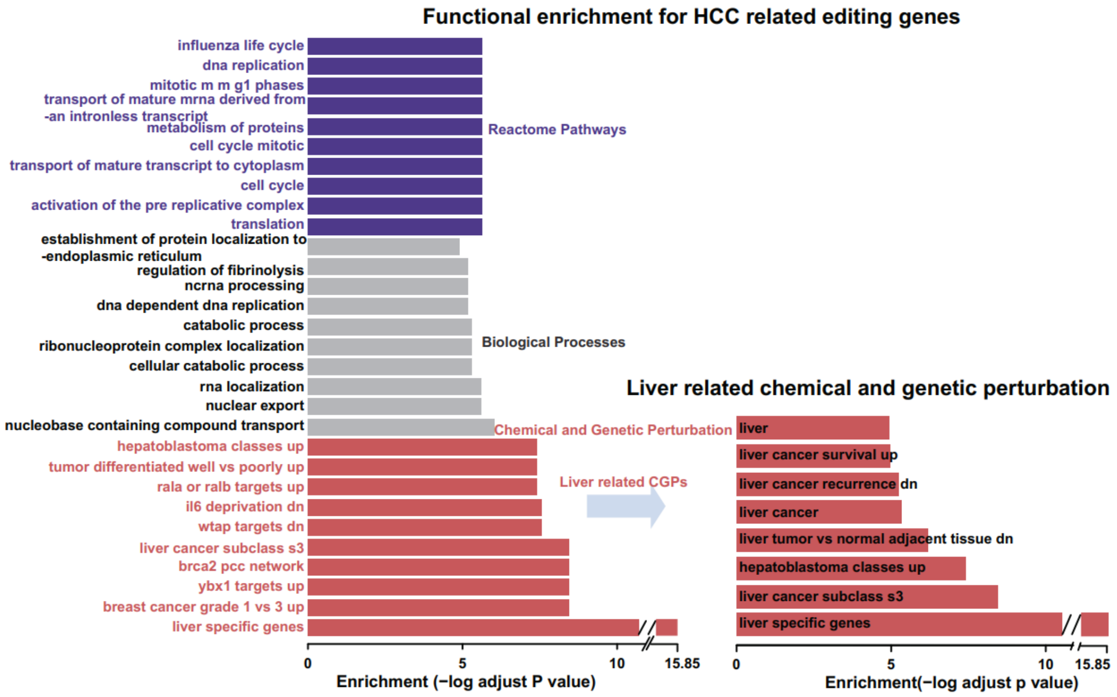

Supplementary Figure S2. Functional enrichment for genes that contain HCC related editing sites. The functional genesets were downloaded from MsigDB, including Reactome pathways, Biological processes and Chemical and Genetic Perturbations.

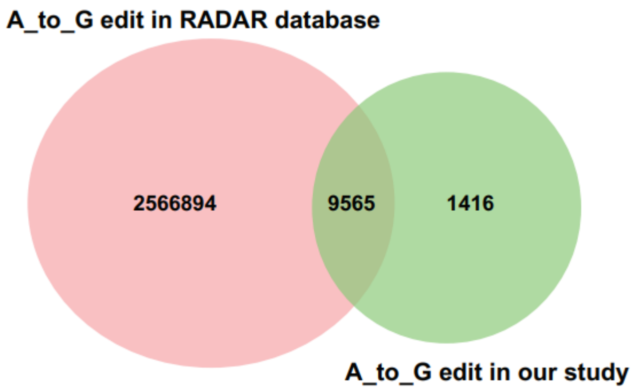

Supplementary Figure S3. The overlap of A-to-G editing sites between our study and RADAR database.

Supplementary Tables

Supplementary Table S1. Data retrieval summary

Data types	Data sources	Sample information
1) Pair-end RNA-seq BAM files for identifying editing sites	dbGap originated from TCGA;<br>https://portal.gdc.cancer.gov/	373 human HCC samples and 50 normal liver samples
2) Validated Pair-end RNA-seq FASTQ files	ArrayExpress, E-MTAB-4052;<br>https://www.ebi.ac.uk/arrayexpress/	3 human normal liver samples and 2 Huh7 cell lines
3) DNA somatic mutation maf files	TCGA; https://portal.gdc.cancer.gov/	373 HCC cancer samples
4) Gene expression HTseq counts data	TCGA; https://portal.gdc.cancer.gov/	374 HCC cancer samples and 50 normal liver samples
5) Clinical information data	TCGA; https://portal.gdc.cancer.gov/	377 HCC cancer samples
6) SNP annotations	dbSNP version 137 and the 1000 genomes project	/
7) Gene annotation of 3’UTR, 5’UTR, CDS and intron regions	UCSC table browser (http://genome.ucsc.edu/cgi-bin/hgTables)	/
8) Functional annotation gene sets	MsigDB database (http://software.broadinstitute.org/gsea/msigdb)	/
9) OGs and TSGs	From a previous study(PMID: 24220575)	/

Supplementary Table S2. Functional enrichment of gene datasets for genes with HCC related RNA editing events.

See **Supplementary Table S2**

Supplementary Table S3. HCC related RNA editing sites located in gene regions.

See **Supplementary Table S3**

Supplementary Table S4. HCC related RNA editing sites with functional consequences.

See **Supplementary Table S4**

Supplementary Table S5. Top 3 affected miRNAs and their target genes that were influenced by HCC related RNA editing.

miRNA	Gene_name	RNA_editing	*E* in reference	*E* in edited	MiR-tar relationship
**hsa-miR-17-3p**	**BORCS7-ASMT;AS3MT**	**chr10;102901274**	**NA**	**-15.95**	**gain**
**hsa-miR-17-3p**	**METTL7A**	**chr12;50930856**	**NA**	**-20.16**	**gain**
**hsa-miR-17-3p**	**TMOD3**	**chr15;51915004**	**NA**	**-19.23**	**gain**
**hsa-miR-17-3p**	**C15orf38-AP3S2;ARPIN**	**chr15;89897976**	**NA**	**-19.79**	**gain**
**hsa-miR-17-3p**	**CPPED1**	**chr16;12660856**	**NA**	**-20.11**	**gain**
**hsa-miR-17-3p**	**PEX13**	**chr2;61050759**	**NA**	**-20.36**	**gain**
**hsa-miR-17-3p**	**DCAF16**	**chr4;17801981**	**NA**	**-18.45**	**gain**
**hsa-miR-17-3p**	**DCAF16**	**chr4;17802039**	**NA**	**-16.31**	**gain**
**hsa-miR-17-3p**	**HINT1**	**chr5;131162260**	**NA**	**-17.38**	**gain**
**hsa-miR-17-3p**	**SPC24**	**chr19;11146013**	**-21.18**	**NA**	**loss**
**hsa-miR-17-3p**	**IYD**	**chr6;150403007**	**-17.33**	**NA**	**loss**
**hsa-miR-20b-3p**	**PRIM2**	**chr6;57646487**	**NA**	**-20.22**	**gain**
**hsa-miR-20b-3p**	**METTL7A**	**chr12;50930856**	**-17.64**	**NA**	**loss**
**hsa-miR-20b-3p**	**TMOD3**	**chr15;51915004**	**-18.29**	**NA**	**loss**
**hsa-miR-20b-3p**	**C15orf38-AP3S2;ARPIN**	**chr15;89897976**	**-15.55**	**NA**	**loss**
**hsa-miR-20b-3p**	**CPPED1**	**chr16;12660856**	**-16.34**	**NA**	**loss**
**hsa-miR-20b-3p**	**PEX13**	**chr2;61050759**	**-18.18**	**NA**	**loss**
**hsa-miR-20b-3p**	**DCAF16**	**chr4;17801981**	**-16.84**	**NA**	**loss**
**hsa-miR-593-3p**	**MDM4**	**chr1;204557576**	**-19.02**	**NA**	**loss**
**hsa-miR-593-3p**	**MDM4**	**chr1;204557582**	**-19.02**	**NA**	**loss**
**hsa-miR-593-3p**	**C12orf49**	**chr12;116714628**	**-19.38**	**NA**	**loss**

**hsa-miR-593-3p**	**C15orf38-AP3S2;ARPIN**	**chr15;89897322**	**-18.96**	**NA**	**loss**
**hsa-miR-593-3p**	**ZNF566**	**chr19;36445654**	**-18.96**	**NA**	**loss**
**hsa-miR-593-3p**	**MAVS**	**chr20;3869788**	**-18.96**	**NA**	**loss**
**hsa-miR-593-3p**	**CXorf56**	**chrX;119539365**	**-18.96**	**NA**	**loss**

Note: *E* refers to the binding energy of miRNA and target calculated by miRanda and "NA" means that the miRNA-target relationship was not identified by current threshold.

MiR-Tar means the relationships between miRNA and target sequences.

Supplementary Table S6. Functional enrichment of gene datasets for HCC related RNA editing genes with functional consequences.

See **Supplementary Table S6**

Supplementary Table S7. Different thresholds to identify editing sites affecting miRNA target relationships.

**Thresholds**	**≥0**	**≥1**	**≥3**	**≥5**	**≥7**	**≥9**	**≥10**
Number of editing sites affect MiR-Tar	8425	7651	6081	4497	2942	1784	1356
Number of HCC related editing affect MiR-Tar	173	173	143	92	60	37	26
Number of HCC related editing with consequence	186	186	157	108	78	56	46
Enrichment of oncogenic genes for HCC related editing with consequence	*p* =0.03* (n=7)	*p* =0.03* (n=7)	*p* =0.05* (n=6)	*p* =0.02* (n=6)	*p* =0.003* (n=6)	*p* =0.03* (n=4)	*p*=0.07 (n=3)

Note: The threshold means the smallest numbers of miRNA-target pairs were changed by RNA editing events. In the main text, we use the threshold of 10 (the last column).

MiR-Tar means the relationships between miRNA and target sequences.

* refers to *p*-value <0.05
